# Epigenetic dysregulation of secreted frizzled-related proteins in myeloproliferative neoplasms complements the JAK2V617F-mutation

**DOI:** 10.1186/1868-7083-4-12

**Published:** 2012-08-31

**Authors:** Karla Bennemann, Oliver Galm, Stefan Wilop, Claudia Schubert, Tim H Brümmendorf, Edgar Jost

**Affiliations:** 1 Clinic for oncology, hematology and stem cell transplantation, Universitätsklinikum Aachen, RWTH Aachen, Pauwelsstraße 30, Aachen, 52074, Germany

**Keywords:** DNA hypermethylation, MPN, SFRP, Tumor suppressor gene, JAK2

## Abstract

**Background:**

Secreted frizzled-related proteins (*SFRPs*) are antagonists of the Wnt signaling pathway, which plays a central role in stem cell maintenance and differentiation of stem cells and hematopoietic progenitors. Epigenetic downregulation of *SFRPs* by promoter hypermethylation has been described to be involved in the pathogenesis of hematopoietic malignancies. There is an association between aberrant Wnt signaling and the established cancer stem cell concept. In contrast to *BCR-ABL1*-positive chronic myeloid leukemia CML, *BCR-ABL1*-negative myeloproliferative neoplasms (Ph^-^MPN) are characterized by the frequent occurrence of an autoactivating mutation in the *JAK2* tyrosine kinase (*JAK2V617F*) or other mutations in the JAK-STAT pathway. However, pathogenetic mechanisms of *JAK2* mutated or unmutated Ph^-^MPN remain not completely understood. We determined the promoter methylation status of *SFRP-1, -2, -4*, and -*5* in 57 MPN patient samples by methylation-specific polymerase chain reaction (PCR) (MSP). *JAK2V617F* was assessed by allele-specific PCR.

**Results:**

Aberrant methylation among primary MPN samples was 4% for *SFRP-1*, 25% for *SFRP-2*, 2% for *SFRP-4*, and 0% for *SFRP-5*. Hypermethylation of *SFRP-2*, which was the most frequently hypermethylated gene in our study, could not be correlated to any specific MPN subtype. However, we detected a significant correlation between *SFRP-2* methylation and presence of a *JAK2V617F* mutation (*P* = 0.008). None of the 10 CML samples showed any *SFRP*-methylation.

**Conclusions:**

Our data indicate that epigenetic dysregulation of the Wnt signaling pathway is a common event in MPN with aberrant methylation of at least one *SFRP* being detected in 25% of the primary patient samples and in 30% if only accounting for Ph^-^MPN. A significant correlation between *SFRP-2* methylation and presence of *JAK2V617F* in our data supports the hypothesis that epigenetic dysregulation may be a complementary mechanism to genetic aberrations. Aberrant methylation of crucial stem cell maintenance genes seems to contribute to disease pathogenesis in Ph^-^MPN.

## Background

Classical myeloproliferative neoplasms (MPN) according to the WHO classification constitute a group of hematopoietic malignancies and comprise chronic myeloid leukemia (CML), polycythemia vera (PV), essential thrombocythemia (ET), and primary myelofibrosis (PMF). MPN share a common trait of unregulated trilineage myeloproliferation and monoclonal hematopoiesis originating from genetically transformed hematopoietic stem cells. CML is characterized by the well-known, disease-causing translocation t(9;22) with the corresponding fusion gene *BCR-ABL1*. In contrast, *BCR-ABL1*-negative MPN (Ph^-^MPN) present heterogenously in terms of clinical characteristics and cytogenetic aberrations. More than 80% of chromosomal aberrations are imbalanced changes, with trisomies of chromosomes 8 and 9 being the most frequent aberrations. Further cytogenetic findings are chromosomal deletions of 5q, 11q, 13q, 20q, 12p, and Y [1].

The discovery of the *Janus kinase 2 (JAK2) V617F* mutation in 2005 has modified the understanding of the molecular basis of Ph^-^MPN and resulted in a revision of the WHO diagnostic criteria [2-4]. JAK2 is a receptor-associated protein tyrosine kinase signaling via the JAK/signal transducer and activator of transcription (JAK-STAT) pathway which plays a key role in a wide spectrum of cellular processes, including proliferation, survival, and normal function of hematopoietic cells. *JAK2V617F* results from a somatic G to T mutation at nucleotide 1849 in exon 14 with consecutive Val617Phe substitution. The mutation affects the non-catalytic pseudo-kinase domain of the JAK2 protein and is responsible for the loss of autoregulatory functionality with constitutive signaling and subsequent hypersensitivity to hematopoietic growth factors. *JAK2V617F* is by far the most prevalent mutation in Ph^-^MPN with this point mutation being detected in more than 95% of patients with PV, 50% of patients with ET, and 50% of patients with PMF [2,5]. Despite detection of *JAK2V617F* in other myeloid diseases, *JAK2V617F* exhibits a broad specificity to patients with myeloid neoplasms. In addition to the *JAK2V617F* mutation, pathogenesis of the MPN has evolved from a simple to a complex model with a number of novel mutations that have been described in chronic or blast-phase Ph^-^MPN. The most prevalent are mutations in *JAK2 exon 12*, *Myeloproliferative Leukemia Virus* (*MPL*), *TET oncogene family member (TET2)*, *Additional Sex Combs-like 1 (ASXL1)*, *Casitas B-lineage lymphoma proto-oncogene (CBL)*, *Isocitrate dehydrogenase (IDH)1 and 2*, and *IKAROS family zinc finger 1 (IKZF 1)*[6]. However, questions remain concerning the initial disease-causing event in Ph^-^MPN, the development of phenotypic distinct diseases and the mechanisms leading to transformation in acute myeloid leukemia (AML). There is increasing evidence that epigenetic alterations might contribute to the pathogenetic and phenotypic variety of MPN [7].

Epigenetic alterations have been shown to play an important role in tumorigenesis [8]. In particular, aberrant methylation of CpG islands within gene promoter regions is associated with transcriptional inactivation and represents an important mechanism of gene silencing in the pathogenesis of hematopoietic malignancies [9,10]. Epigenetic disturbances are postulated to be a complementary or alternative mechanism to genetics [11,12]. Aberrant CpG island methylation in MPN has been reported for *p15* and *p16* (cell cycle regulation), as well as for *retinoic acid receptor beta 2 (RARβ2)* and *Abelson* (*ABL*) [13,14]. Furthermore, the *stromal cell-derived factor-1 (SDF-1) receptor CXCR4* is abnormally downregulated in CD34-positive hematopoietic progenitor cells that constitutively circulate in PMF patients. Functional reconstitution with demethylating agents in combination with the histone deacetylase (HDAC) inhibitor results in reduction of *in vitro* generated *JAK2V617F* mutated cells [15]. Hypermethylation of the JAK-STAT inhibitor *suppressor of cytokine signaling* (*SOCS) 1* has been shown by independent groups and downregulation of *SOCS-3* as well as *src homology region 2 domain-containing phosphatase 1* (*SHP1*) has been found to be associated with promoter methylation in MPN patients [12,16] Further evidence of involvement of epigenetic dysregulation in MPN pathogenesis evolve from the findings of loss-of-function mutations in *enhancer of zeste homolog 2* (*EZH2*), *ASXL1* and *TET2* with resulting deregulation in both DNA methylation and chromatin structure [17].

To gain additional information about the prevalence and pathogenetic relevance of epigenetic changes as complementary or alternative mechanism in the pathogenesis in MPN, we determined the promoter methylation status of secreted frizzled-related proteins (*SFRPs*). *SFRP-1, -2, -4*, and -*5* possess a CpG island in the promoter region and are antagonists of the Wnt signaling pathway. Consistent with the key functions of the Wnt pathway in stem cell maintenance and differentiation of hematopoietic progenitors, epigenetic downregulation of SFRPs has been described in hematopoietic malignancies [18,19].

## Results

### Methylation analysis of SFRP-1, -2, -4 and -5

**Figure 1:**
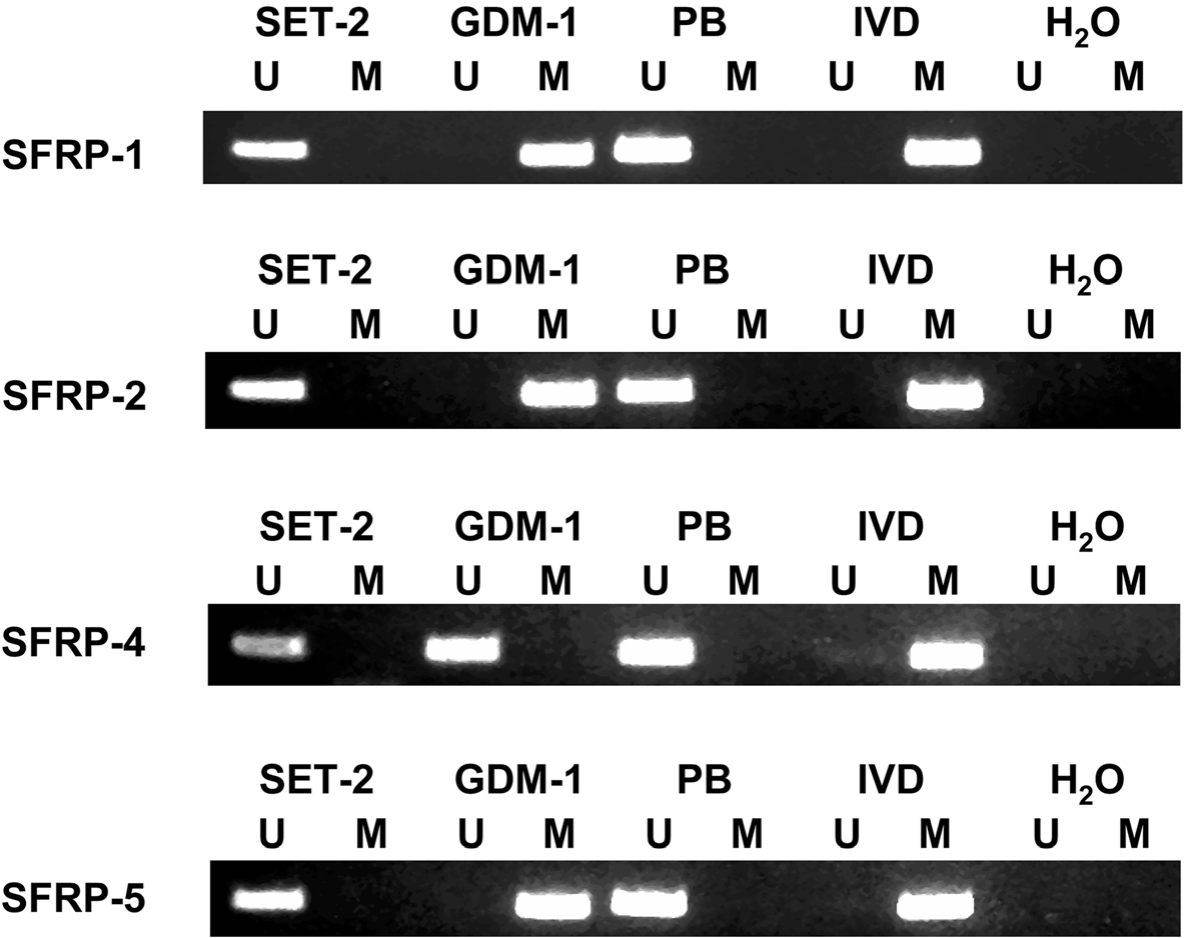
**MSP analysis of the four SFRP genes in MPN-derived cell lines and normal peripheral blood (PB).***In vitro* methylated DNA (IVD) and water served as controls. *Lane U*, amplified product with primers recognizing unmethylated *SFRP-1, -2, -4*, and -*5* sequences. *Lane M*, amplified product with primers recognizing methylated *SFRP-1, -2, -4*, and -*5* sequences.

**Figure 2:**
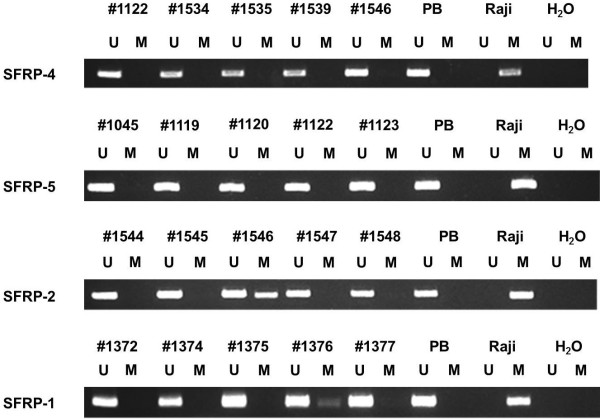
**Representative MSP analysis of the four SFRP genes in MPN patient samples.** DNA from Raji cells and water served as controls. *Lane U*, amplified product with primers recognizing unmethylated *SFRP-1, -2, -4*, and -*5* sequences. *Lane M*, amplified product with primers recognizing methylated *SFRP-1, -2, -4*, and -*5* sequences. Patient sample #1546 methylated at the *SFRP-2* promoter, patient sample #1376 methylated at the *SFRP-1* promoter.

We first analyzed the methylation status of *SFRP-1, -2, -4*, and -*5* promoter regions by MSP in the human MPN-derived cell lines SET-2 and GDM-1. Methylation was found for *SFRP-1, -2*, and -*5* in GDM-1 cells (Figure 1). None of the four genes showed aberrant methylation in SET-2 cells. Methylation-associated gene inactivation of *SFRP-1* and -*2* in AML cell lines has been published previously [18]. Owing to the functional importance of the Wnt pathway in several hematopoietic malignancies, we then investigated the methylation status of the *SFRP* promoter regions in PB and BM from 57 primary MPN patient samples obtained at diagnosis or during follow-up. Table 1 gives an overview of the characteristics of the patient cohort. Representative MSP-results for *SFRP-1, -2, -4*, and -*5* are shown in Figure 2.

**Table 1:** Characteristics of the patient cohort

	**JAK2**		**Age**	**Gender**		**WBC (10**^**9**^**/L)**	**Hb (g/L)**	**Plt (10**^**9**^**/L)**	**Blasts (%)**	**LDH (U/L)**
	**mut**	**wt**		**m**	**f**					
CML (*n* = 10)	0	10	55	9	1	81.3	107	219	1	599
PV (*n* = 10)	8	2	56	4	6	12.4	133	370	0	259
ET (*n* = 10)	5	5	69	3	7	8.2	130,5	770	0	267
MF (*n* = 27)	14	13	67	12	15	10.1	104,5	376	0,5	431
Total	27	30	66	28	29	11.7	114	338	0	369

Median values for age, WBC, Hb, Plt, Blasts, LDH. CML, Chronic myeloid leukemia, ET, essential thrombocythemia; Hb, hemoglobin; LDH, lactate dehydrogenase; MF, myelofibrosis; mut, positive for JAK2V617F; plt, platelet count; PV, polycythemiavera; WBC, white blood cell count; wt = negative for JAK2V617F.

The frequency of aberrant methylation among primary MPN patient samples was 4% (2/57) for *SFRP-1*, 25% (14/57) for *SFRP-2*, 2% (1/57) for *SFRP-4*, and 0% (0/57) for *SFRP-5*. Aberrant methylation of at least one of the *SFRP* genes was detected in all Ph^-^MPN subtypes. In contrast, no methylation of any of the *SFRPs* has been detected in CML samples. Among the Ph^-^MPN subtypes we found methylation of any SFRP in 30% (3/10) PV-patient samples, 40% (4/10) ET-patient samples, and 26% (7/27) MF-patient samples. Regarding the rare methylation of *SFRP-4* and -*5*, only *SFRP-1* and -*2* were considered for further statistical analyses. We did not see any significant association between *SFRP-2* methylation and MPN subtype. However, there was an association of *SFRP-1* hypermethylation and diagnosis of ET (2/2 *SFRP-1* promoter methylations in ET patients, *P* = 0.02). We could not show any correlation between *SFRP-2* methylation and platelet count, peripheral blast cell count, red blood cell count, white blood cell count (WBC), or lactate dehydrogenase in PB. Furthermore, a history of cytostatic therapy did not influence *SFRP-2* methylation frequency in our patient cohort.

### JAK2 mutation analysis

**Figure 3:**
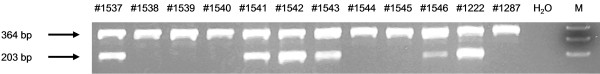
**Representative JAK2 mutation analysis of MPN patient samples by allele-specific PCR.** The 203 base pairs (bp) product is specific for the mutant allele, whereas the 364 bp product amplifying both mutant and wild-type alleles serves as an internal PCR control. DNA from a healthy donor (#1287) was used as a negative control. DNA from a patient with homozygous *JAK2V617F* mutation served as a positive control (#1222, mutation verified by sequencing). In a dilution series of DNA from a patient with a homozygous JAK2V617F mutation with normal DNA, the sensitivity of the PCR could be shown to be at least 1/64. M = DNA ladder. Patient samples #1537, #1541, #1542, #1543, and #1546 carry a *JAK2V617F* mutation.

Using allele-specific PCR, 47% (27/57) of the patients showed a signal corresponding to the *JAK2* G >T mutation. A total of 50% (5/10) ET and 80% (8/10) PV patients had a *JAK2* mutation. Among the MF samples, 52% (14/27) carried the mutation. In accordance with expectations we did not detect *JAK2V617F* in CML patient samples. We could not show any correlation between *JAK2V617* mutation and laboratory findings. Importantly, there was no association between previous cytostatic therapy and presence of JAK2V617F. Representative results of the allel-specific PCR are shown in Figure 3.

### Epigenetic and genetic changes

Of the two patient samples methylated at the *SFRP-1* promoter, one carried the mutation whereas the other one was wild-type *JAK2*. In contrast, of the 14 patients with aberrant methylation of *SFRP-2*, 78.6% (11/14) were positive for the *JAK2V617F* mutation whereas 21.4% (3/14) showed wild-type *JAK2*. Thus, there is a significant correlation of *SFRP-2* methylation occurring in association with a mutated status at *JAK2* (*P* = 0.008). Table 2 gives a view of concomitant *JAK2* mutation and *SFRP-2* promoter methylation in patients with MPN.

**Table 2:** Concomitant JAK2 mutation and SFRP-2 hypermethylation in MPN patients

	**CML (*n *= 10)**			**PV (*n *= 10)**		**ET (*n *= 10)**		**MF (*n *= 27)**	
SFRP-2	U	M	U	M	U	M	U	M
JAK2, wt	10	0	1	1	4	1	12	1
JAK2V617F	0	0	6	2	2	3	8	6

CML, chronic myeloid leukemia; ET, essential thrombocythemia; M, methylated; MF, myelofibrosis; PV, polycythemiavera; U, unmethylated; wt = negative for JAK2V617F.

## Discussion

To the best of our knowledge, no data have been published so far about aberrant CpG island hypermethylation of *SFRP-1, -2, -4*, and -*5* in the context of *JAK2V617F* in Ph^-^MPN.

In contrast to CML, a number of novel mutations have been described in *BCR-ABL1*-negative MPN. Among those are mutations involving *JAK2*, *MPL*, *TET2*, *ASXL1*, *CBL*, *IDH*, and *IKZF1*[17,20]. Wnt signaling plays an important role in stem-cell self-renewal as well as in differentiation and proliferation of hematopoietic progenitor cells. Recently, it has been reported that Wnt signaling could be one of the mechanisms that shape a niche in the bone marrow supportive of hematopoiesis [21]. Since aberrant activation of the Wnt pathway has been demonstrated to contribute to leukemogenesis and until now, in contrast to solid tumors, no activating mutations in the genes of the Wnt pathway have been described in myeloid or lymphatic malignancies, we investigated epigenetic regulation of *SFRP-1, -2, -4*, and -*5* as negative regulators of Wnt signaling. Our data indicate that CpG island hypermethylation of *SFRP* genes is a frequent event in Ph^-^MPN with aberrant methylation of at least one of the four genes being detected in 30% of the primary patient samples. Compared to the *SFRP* methylation patterns in other hematopoietic malignancies, aberrant promoter methylation in our study occurred less frequently than in AML [18]. We could show a preferential hypermethylation of *SFRP-1* (4%) and of *SFRP-2* (25%), in particular. This is in contrast to the methylation frequency of *SFRP* genes in acute lymphatic leukemia (ALL) [22], where a more balanced methylation frequency of the four genes could be shown, but confirms findings in AML showing a preferential hypermethylation of *SFRP-1* (29%) and *SFRP-2* (19%).

We could show a significant association of *SFRP-1* hypermethylation and diagnosis of ET. Regarding the limited number of patient samples methylated at the *SFRP-1* promoter (2/57), those results require further confirmation. Furthermore, a study in 52 ET samples did not find any evidence of hypermethylation of soluble Wnt inhibitors [23], whereas our analysis, also using MSP and the same primer sequences, revealed a rate of 40% of ET samples being methylated.

In contrast to *SFRP-1*, *SFRP-2* hypermethylation in our study occurred in all Ph^-^MPN subtypes without any association with a specific entity. However, there was a significant correlation between aberrant *SFRP-2* promoter methylation and the presence of *JAK2V617F*.

The autoactivating *JAK2V617F* mutation is responsible for constitutive signaling of the JAK2 protein with subsequent hypersensitivity to hematopoietic growth factors [3]. When comparing the incidence of the *JAK2V617F* mutation and the frequency among the MPN subtypes with published data, the relative distribution (80% PV, 50% ET, 52% MF) we obtained in our study is in accordance with previous reports [5]. Limited data are available about epigenetic disturbances in MPN. Increasing evidence suggests that loss-of-function mutations in genes involved in epigenetic regulation, *TET2*, *ASXL1*, and *EZH2*, as well as JAK2V617F-mediated phosphorylation of *protein Arg N-methyltransferase* (*PRMT5)* may play a role in disease pathogenesis, either as ‘pre-*JAK2* events’ or occurring in late phase disease [17,24-26].

Aberrant CpG island methylation has been reported for *ABL*, *p14*, *p15*, *p16*, *RARß2*[13], and *SDF1* receptor *CXCR4*[15]. SOCS proteins are the most thoroughly studied inhibitors of JAK-STAT pathways. Silencing of *SOCS-1* and -*3* in association with promoter hypermethylation has been reported by different groups [12,16,27]. However, Capello *et al*. reported that *SOCS-3* methylation was more frequent among *JAK2V617F*-negative patients. Nevertheless, the results suggest that epigenetic inactivation seems to play a role in Ph^-^MPN. Epigenetics might be a complementary or alternative mechanism to the *JAK2V617F* mutation in the pathogenesis of Ph^-^MPN, leading to dysregulation of JAK-STAT signal transduction and thus to growth factor hypersensitivity. Our findings are in accordance with this hypothesis showing a trend towards an association between *JAK2V617F* and *SFRP-2* hypermethylation, thus, an association between genetic and epigenetic aberrations. Interesting in this context is the comparison of Ph^-^MPN with CML. The identified disease-causing mutation in CML which codes for a fusion-protein with tyrosine kinase activity provides a potential therapeutic target. The clinical success of the tyrosine kinase inhibitor imatinib suggests that the mutation might be one of the main events leading to the pathogenesis of CML. However, regarding the reported Wnt pathway activation in CML cancer stem cells [28] and the reported correlation of *SFRP-1* promoter methylation and resistance to imatinib [29] the impact of hypermethylation-associated gene silencing in CML in contrast to Ph^-^MPN has to be further elucidated. According to our study, *SFRP* promoter hypermethylation in CML seems to be an uncommon event.

## Conclusion

Despite the discovery of the nearly disease specific *JAK2V617F*-mutation and its capacity to induce myeloproliferative disease-like phenotypes in murine models [30], additional genetic or epigenetic events are thought to play an important role in disease initiation. Furthermore, development of three distinct entities from one known mutation has to be explored. Since our patient cohort only consisted of 57 samples and was heterogenous for prior treatment regimens, no definitive conclusion can be retained from these data neither regarding the pathogenetic interrelationship of genetic and epigenetic events, nor the phenotypic differences between Ph^-^MPN subtypes or resulting treatment options in a group of diseases with allogenic stem cell transplantation being the only curative concept. Tyrosine kinases are essential therapeutic targets in several diseases and development of inhibitors of JAK2 are being tested in clinical studies. Targeting the epigenome might be a worthwhile effort to complement existing or newly developed therapeutic strategies [6].

## Methods

### Human tissue samples

All samples from patients were collected after informed consent was given. Thirty-four peripheral blood (PB) and 23 bone marrow (BM) specimens were obtained during routine clinical assessment between 1996 and 2007 of 57 adult patients with MPN without any evidence for leukemic transformation. According to standard criteria [31], patients were classified as having CML (*n* = 10), ET (*n* = 10), PV (*n* = 10), and MF (*n* = 27). The collection of patient samples for analysis of genetic and epigenetic changes was approved by the local ethics committee. DNA extraction was performed from unselected cells from BM, when available, or PB. Additionally, we examined the methylation status of MPN-derived cell lines GDM-1 and SET-2.

### Methylation analysis of SFRP-1, -2, -4, and -5

BM and PB mononuclear cells were isolated by density gradient centrifugation according to standard procedures prior to further analysis. Assessment of peripheral blood mononuclear cells is appropriate regarding the high sensitivity of the applied MSP methods (0.1%) [32]. For the DNA isolation the QIAmp® DNA Mini Kit (Quiagen, Hilden, Germany) was used according to the manufactures instructions. The methylation status of *SFRP-1, -2, -4*, and -*5* was investigated by MSP. Approximately 1 μg of DNA was chemically modified by sodium bisulfit treatment prior to MSP. This assay converts all unmethylated cytosins to uracil but leaving the methylated cytosins unaffected. Subsequently, amplification with primers specific for methylated *vs*. unmethylated DNA was performed [32]. MSP primer sequences and reaction conditions for *SFRP-1*, *-2*, *-4*, and -*5* have been described previously [33]. The primer specific reaction temperatures were 60°C for *SFRP-1* and -*2*, 62°C for *SFRP-4* and 63°C for *SFRP-5*. For every MSP reaction, *in vitro* methylated DNA (IVD) or the Burkitt lymphoma cell line Raji served as positive and normal PB from healthy volunteers served as negative controls. PCR products were separated on 2.5% agarose gels and visualized by ethidium bromide staining.

### JAK2 mutation analysis

The mutational status of the *JAK2* gene was assessed by allele-specific PCR. Primers and reaction conditions used were previously described [2]. After amplification, the presence of a *JAK2V617F* mutation was visualized under UV light on 2% agarose gels after ethidium bromide staining. In a 1:2 dilution series of DNA from a patient with a homozygous *JAK2V617F* mutation with normal DNA, the sensitivity of the PCR was shown to be at least 1/64. Direct sequencing of the samples mutated by allele specific PCR confirmed the JAK2V617F mutation in most of the patients and is a proof for a sufficient proportion of clonal cells in the starting material (data not shown). DNA from a patient with a homozygous *JAK2V617F* mutation was used as a positive control. DNA from a healthy donor was used as a negative control for every PCR reaction.

### Statistical methods

Correlations between variables were analyzed using the Fisher’s exact two-sided test and the two-sided Student’s *t*-test, respectively. All calculations were performed using the SAS statistical software version 9.1.3.

## Competing interests

The authors declare that they have no competing interests.

## Authors’ contributions

KB carried out the molecular genetic analyses, participated in the design of the study, and drafted the manuscript. CS also carried out molecular genetic analyses and made a significant contribution to the general supervision of the research group. EJ, TB, and OG conceived of the study, participated in design and organization of the study, and helped to draft the manuscript. SW performed the statistical analyses. All authors read and approved the manuscript. All authors gave final approval to version to be published.
